# Positioning Head Tilt in Canine Lysosomal Storage Disease: A Retrospective Observational Descriptive Study

**DOI:** 10.3389/fvets.2021.802668

**Published:** 2021-12-14

**Authors:** Shinji Tamura, Yumiko Tamura, Yuya Nakamoto, Daisuke Hasegawa, Masaya Tsuboi, Kazuyuki Uchida, Akira Yabuki, Osamu Yamato

**Affiliations:** ^1^Tamura Animal Clinic, Hiroshima, Japan; ^2^Neuro Vets Animal Neurology Clinic, Kyoto, Japan; ^3^Veterinary Surgery, Graduate School of Life and Environmental Science, Osaka Prefecture University, Sakai, Japan; ^4^Laboratory of Veterinary Radiology, Nippon Veterinary and Life Science University, Tokyo, Japan; ^5^Laboratory of Veterinary Pathology, Graduate School of Agriculture and Life Science, University of Tokyo, Tokyo, Japan; ^6^Laboratory of Clinical Pathology, Joint Faculty of Veterinary Medicine, Kagoshima University, Kagoshima, Japan

**Keywords:** positioning head tilt, dog, lysosomal storage disease, ceroid lipofuscinosis, GM1 gangliosidosis, GM2 gangliosidosis, nodulus, ventral uvula

## Abstract

Positioning head tilt is a neurological sign that has recently been described in dogs with congenital cerebellar malformations. This head tilt is triggered in response to head movement and is believed to be caused by a lack of inhibition of the vestibular nuclei by the cerebellar nodulus and ventral uvula (NU), as originally reported cases were dogs with NU hypoplasia. We hypothesized that other diseases, such as lysosomal storage diseases that cause degeneration in the whole brain, including NU, may cause NU dysfunction and positioning head tilt. Videos of the clinical signs of canine lysosomal storage disease were retrospectively evaluated. In addition, post-mortem NU specimens from each dog were histopathologically evaluated. Nine dogs were included, five with lysosomal storage disease, two Chihuahuas with neuronal ceroid lipofuscinosis (NCL), two Border Collies with NCL, one Shikoku Inu with NCL, two Toy Poodles with GM2 gangliosidosis, and two Shiba Inus with GM1 gangliosidosis. Twenty-eight videos recorded the clinical signs of the dogs. In these videos, positioning head tilt was observed in seven of nine dogs, two Chihuahuas with NCL, one Border Collie with NCL, one Shikoku Inu with NCL, one Toy Poodle with GM2 gangliosidosis, and two Shiba Inus with GM1 gangliosidosis. Neuronal degeneration and loss of NU were histopathologically confirmed in all diseases. As positioning head tilt had not been described until 2016, it may have been overlooked and may be a common clinical sign and pathophysiology in dogs with NU dysfunction.

## Introduction

The concept of positioning head tilt (PHT) as a neurological sign has recently been established. Dogs with this symptom can turn freely in any direction at their will. The head is in a normal position when static or when the dog walks straight forward. However, the head tilts to the opposite side when the dog turns; when the dog turns to the right, the left head tilt appears. Therefore, the direction of head tilt changes every time the dog turns its head. This neurological sign is believed to occur in response to head movement and is caused by a lack of inhibition of the vestibular nuclei by the cerebellar nodulus and ventral uvula (NU) (1, 2). The original documented cases were three dogs with presumptive NU hypoplasia (1). Recently, PHT was observed in a dog with gliomatosis cerebri affecting NU (3–5). We hypothesized that other diseases, such as lysosomal storage diseases (LSDs) that cause degeneration in the whole brain, including the cerebellum, may also cause NU dysfunction and PHT. To verify this hypothesis, we reevaluated the clinical signs and histopathological changes in the NU of dogs with LSDs that we had previously encountered retrospectively.

## Materials and Methods

This is a retrospective, observational, descriptive study. Therefore, live animals were not used in this study, and ethical approval is not required due to their nature. However, all owners of the cases, including this study, received informed consent and had approved that their dogs and data would be used for academic research.

### Cases and Data Collection

Stored videos recording neurological signs of canine cases with definitively diagnosed LSDs were collected from three veterinary clinics. Clinical data of those cases were collected, including breed, sex, age of clinical onset, age of death, method of definitive diagnosis, genetic mutation, magnetic resonance imaging (MRI), and tissue specimens of the cerebellum, including NU (Table 1).

**Table 1:** Summary of the cases studied.

**Case**	**Disease**	**Breed/sex**	**Type**	**Onset (months)**	**Age when videos are available (months)**	**Death (months)**	**Diagnosis**	**Gene**	**Mutation**
1	NCL	CH/M	LJ	18	19, 22, 23	24	G, P	*MFSD8*	*MFSD8*:c.843delT chr19:13,010,759delT^*^
2	NCL	CH/M	LJ	16	18, 21	23E	G, P	*MFSD8*	*MFSD8*:c.843delT chr19:13,010,759delT^*^
3	NCL	BC/M	LJ	16	16	25E	G, P	*CLN5*	*CLN5*:c.619C>T chr22:30,574,637C>T^*^
4	NCL	BC/F	LJ	20	23	26	G	*CLN5*	*CLN5*:c.619C>T chr22:30,574,637C>T^*^
5	NCL	Shikoku/M	A	32	35, 44, 46, 47, 49, 51, 53, 65, 68	68E	P	ND	ND
6	GM2	TP/F	LJ	12	21	21E	G, P	*HEXB*	chr2:57,225,684delG^*^
7	GM2	TP/F	LJ	11	16, 16.5, 17, 17.5, 18, 18.5, 19	23	G, LE, TLC, P	*HEXB*	chr2:57,225, 684delG^*^
8	GM1	Shiba/M	J	5	5, 7	11E	G, P	*GLB1*	chr23:3,796, 317delC^*^
9	GM1	Shiba/F	J	6	6, 9	12	G	*GLB1*	chr23:3,796, 317delC^*^

*NCL, neuronal ceroid lipofuscinosis; GM2, GM2 gangliosidosis; GM1, GM1 gangliosidosis; CH, Chihuahua; BC, Border Collie; TP, Toy Poodle; M, male; F, female; LJ, late-juvenile; A, adult; J, juvenile; E, euthanasia; G, genetic test; P, pathology; LE, leukocyte enzyme activity; TLC, thin-layer chromatography; ND, not determined*.

* *CanFam 3.1*.

### Included Cases

Nine dogs were included in this study: two Chihuahuas with neuronal ceroid lipofuscinosis (NCL) (Cases 1 and 2), two Border Collies with NCL (Cases 3 and 4), one Shikoku Inu with NCL (Case 5), two Toy Poodles with GM2 gangliosidosis variant 0 (Sandhoff disease) (Cases 6 and 7), and two Shiba Inus with GM1 gangliosidosis (Cases 8 and 9). All affected dogs were definitively diagnosed by pedigree analysis, genetic testing, lysosomal enzyme activity, thin-layer chromatography, or histopathology (see individual references and Table 1). All dogs with each type of LSD included in this study have been previously published, and detailed clinical, imaging, and pathological findings and methodologies of various tests should be referred to in the literature (6–21). Clinical characteristics of each type of LSD are summarized below.

#### NCL in Chihuahuas

Clinical signs, including behavioral abnormalities such as signs of morbid fear or hyperacusis, visual impairment, and ataxia, began at 16–18 months of age and died of neurological deterioration at 23–24 months. This disease is an inherited disease characterized by lipopigment deposition in neurons and other cells of the body (6–9). This disease is caused by a mutation in the ceroid lipofuscinosis neuronal 7 (*CLN7*/*MFSD8*) gene (7). Detailed clinical, imaging, histological, and molecular characteristics have previously been reported (7–9).

#### NCL in Border Collies

Clinical signs, including behavioral abnormalities, began at 15–20 months of age and worsened progressively, with motor dysfunction, visual impairment, myoclonus/myoclonic seizure appearing at 19–23 months, and lethargy to death at 23–32 months. This disease is an inherited disease characterized by lipopigment deposition in neurons and other cells of the body. This disease is caused by a mutation in the ceroid lipofuscinosis neuronal 5 (*CLN5*) gene. Detailed clinical, imaging, histological, and molecular characteristics have previously been reported (6, 10, 11).

#### NCL in Shikoku Inus

Clinical signs, including gait abnormalities, ventral flexion of the neck, and postural nystagmus, began at 32 months of age and worsened progressively, with visual impairment appearing at 43 months and lethargy to euthanize at 68 months. This disease is characterized by the deposition of lipopigment in neurons and other cells in the body. This disease is predicted to be an inherited disease, but the causative mutation has not yet been identified. Detailed clinical, imaging, and histological characteristics have previously been reported (12).

#### GM2 Gangliosidosis Variant 0 (Sandhoff Disease) in Toy Poodles

Clinical signs, including motor disorders, ataxia, intention tremor, decreased corneal reflex, and absence of menace response, began at ~9–12 months of age (6, 13, 14). The animals died of neurological deterioration at 18–23 months. This disease is an autosomal recessive deficiency of β-hexosaminidase A and B enzyme activity due to a frameshift mutation of the *HEXB* gene (14). Detailed clinical, imaging, histological, and molecular characteristics have previously been reported (6, 13, 14).

#### GM1 Gangliosidosis in Shiba Inus

Clinical signs, including cerebellar ataxia, began at 5–6 months of age and worsened progressively, with cerebral signs appearing at 7–8 months, atactic abasia or astasia at 9–10 months, and lethargy to death at ~11 months (6, 15). This disease is an autosomal recessive deficiency of β-galactosidase enzyme activity due to a mutation of the *GLB1* gene (16). Detailed clinical, imaging, histological, and molecular characteristics have previously been reported (6, 15–22).

### Evaluation of Videos, MRIs, and Histopathology

All videos stored of these dogs were carefully evaluated to determine whether the dogs showed PHT or not by three experienced veterinary neurologists (ST, YN, and DH) in the council system. The recorded timings of the videos for each case are summarized in Table 1.

MRI of the brain was obtained with a 0.3-Tesla system (Airis II comfort, FUJIFILM Healthcare Corporation, Tokyo, Japan) in Cases 1–7 and 9, and with a 1.5-Tesla system (Visart, Toshiba Corporation, Tokyo, Japan) in Case 8. Sagittal T2-weighted images of these cases were evaluated for cerebellar atrophy by three experienced veterinary neurologists (ST, YN, and DH) in the council system.

Brain tissue specimens of each disease (Cases 1, 3, 5, 7, and 9) were collected at necropsy, and the specimens were prepared by fixed and stored in 10% formalin. Paraffin-embedded tissue sections, including NU prepared by the standard method and stained with hematoxylin and eosin, were evaluated and compared to that of a normal control dog by veterinary neuropathologists (MT, KU).

## Results

### Evaluation of Videos

**Figure 1:**
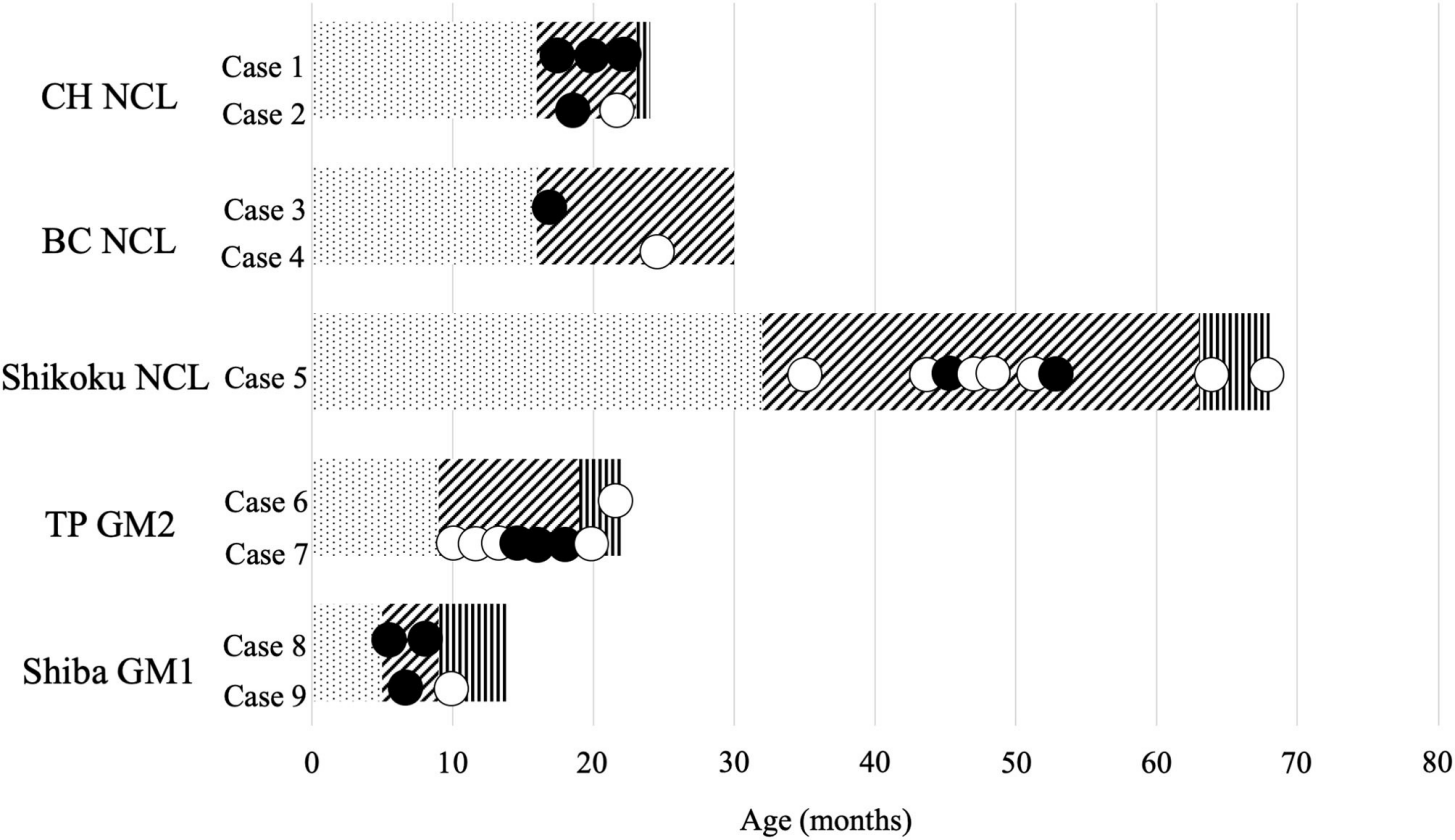
Available videos of canine lysosomal storage diseases. CH, Chihuahua; BC, Border Collie; TP, Toy Poodle; NCL, neuronal ceroid lipofuscinosis; GM2, GM2 gangliosidosis; GM1, GM1 gangliosidosis. The bar graph indicates the approximate stages of disease: dot, before onset; diagonal line, with clinical signs; vertical line, in a lateral recumbent position. Black circle, videos with positioning head tilt observed; white circle, videos without positioning head tilt observed.

**Figure 2:**
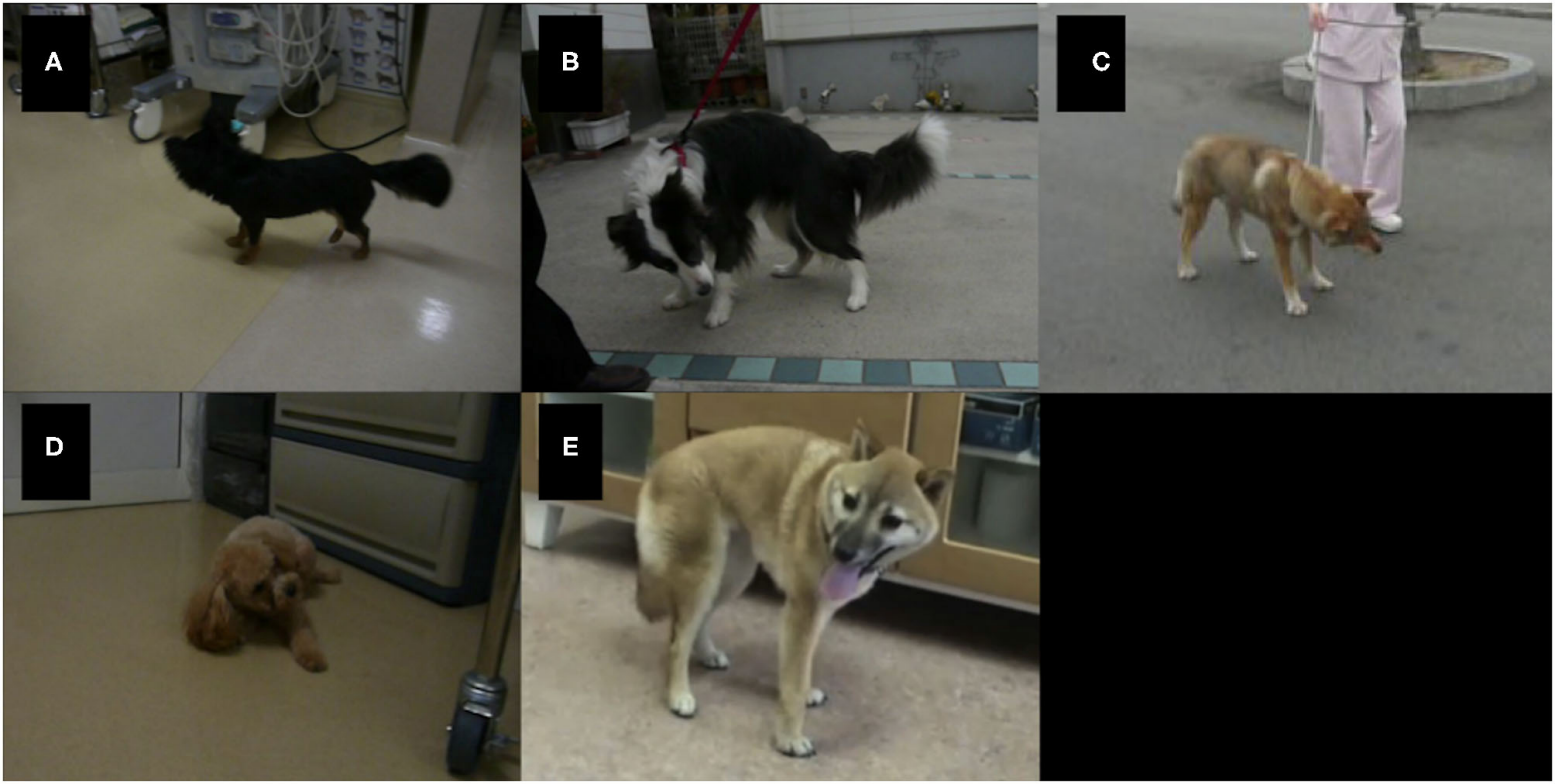
Positioning head tilt observed in dogs with lysosomal storage diseases. The heads of all dogs tilt to opposite sides when turning to one side. **(A)** A Chihuahua with neuronal ceroid lipofuscinosis (NCL) (Case 1, 19 months of age); **(B)** a Border Collie with NCL (Case 3, 16 months of age); **(C)** a Shikoku Inu with NCL (Case 5, 46 months of age); **(D)** a Toy Poodle with GM2 gangliosidosis variant 0 (Sandhoff disease) (Case 7, 18 months of age); **(E)** a Shiba Inu with GM1 gangliosidosis (Case 9, 6 months of age).

Twenty-eight videos that recorded the clinical signs of the nine cases were available. The relationship between the approximate stages of disease (7, 10–13, 17) and the recorded time of each video is summarized in Figure 1. PHT was observed in two Chihuahuas with NCL (Cases 1 and 2), one Border Collie with NCL (Case 3), one Shikoku Inu with NCL (Case 5), one Toy Poodle with GM2 gangliosidosis (Case 7), and two Shiba Inus with GM1 gangliosidosis (Cases 8 and 9). In Case 1 (Chihuahua with NCL), PHT was slightly observed at 19 months of age and was relatively more significant at 22 months. A right-left directed abnormal repetition movement behavior, which elicited PHT every head turn, was observed at 23 months of age. In Case 3 (Border Collie with NCL), PHT was slightly observed at 16 months of age. In Case 5 (Shikoku Inu with NCL), PHT was clearly observed at 46 months of age and was once not observed and was again observed at 53 months. It was unevaluable at 65 and 68 months, as the dog could not maintain the trunk position and recumbency. In Case 7 (Toy Poodle with GM2 gangliosidosis variant 0), PHT was not observed at 17 months of age but was relatively clearly observed at 18.5 months and was unevaluable at 19 months as the dog recumbent. A slight PHT was observed in Case 9 (Shiba Inu with GM1 gangliosidosis) at 6 months of age and was unevaluable at 9 months, as the dog was recumbent. The PHT of these dogs is shown in Figure 2, and the videos are included in Supplementary Videos 1, 2. The PHT in the other cases is summarized in Figure 1.

### MRI Findings of NU

**Figure 3:**
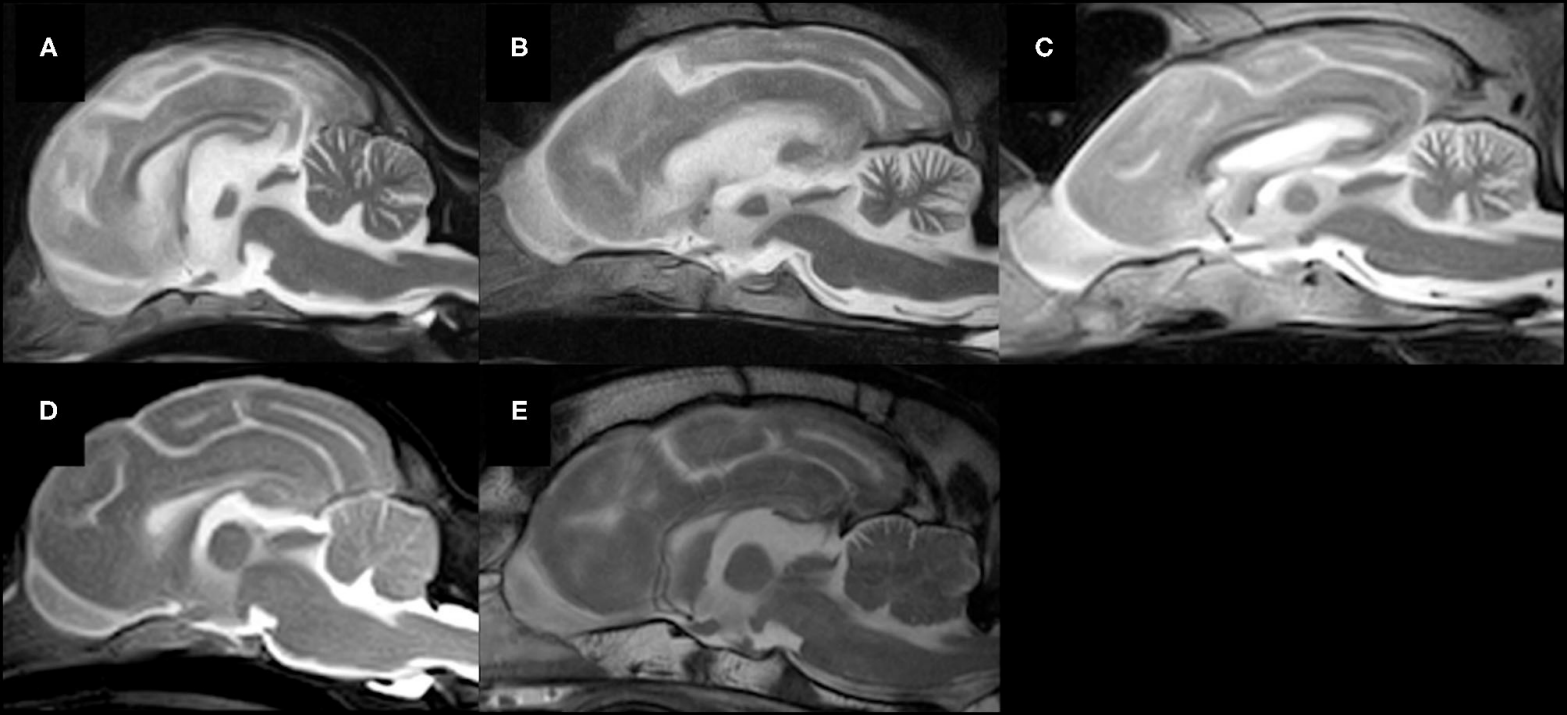
Mid-sagittal T2-weighted MR images of five lysosomal storage diseases. **(A)** A Chihuahua with NCL (Case 1, 19 months of age), **(B)** a Border Collie with NCL (Case 3, 16 months of age), **(C)** a Shikoku Inu with NCL (Case 5, 68 months of age), **(D)** a Toy Poodle with GM2 gangliosidosis variant 0 (Sandhoff disease) (Case 7, 21 months of age), **(E)** a Shiba Inu with GM1 gangliosidosis (Case 8, 11 months of age). Atrophy of the whole brain, including the cerebellum, is observed in all cases.

Widened fissures of the cerebellar vermis and dilated cerebrospinal fluid space between the cerebellum and brain stem, including the fourth ventricle, were observed in all cases on mid-sagittal T2-weighted MR images, indicating cerebellar atrophy (23–25). However, the details of abnormalities by region in the cerebellum were not clear (Figure 3).

### Histopathological Findings of NU

**Figure 4:**
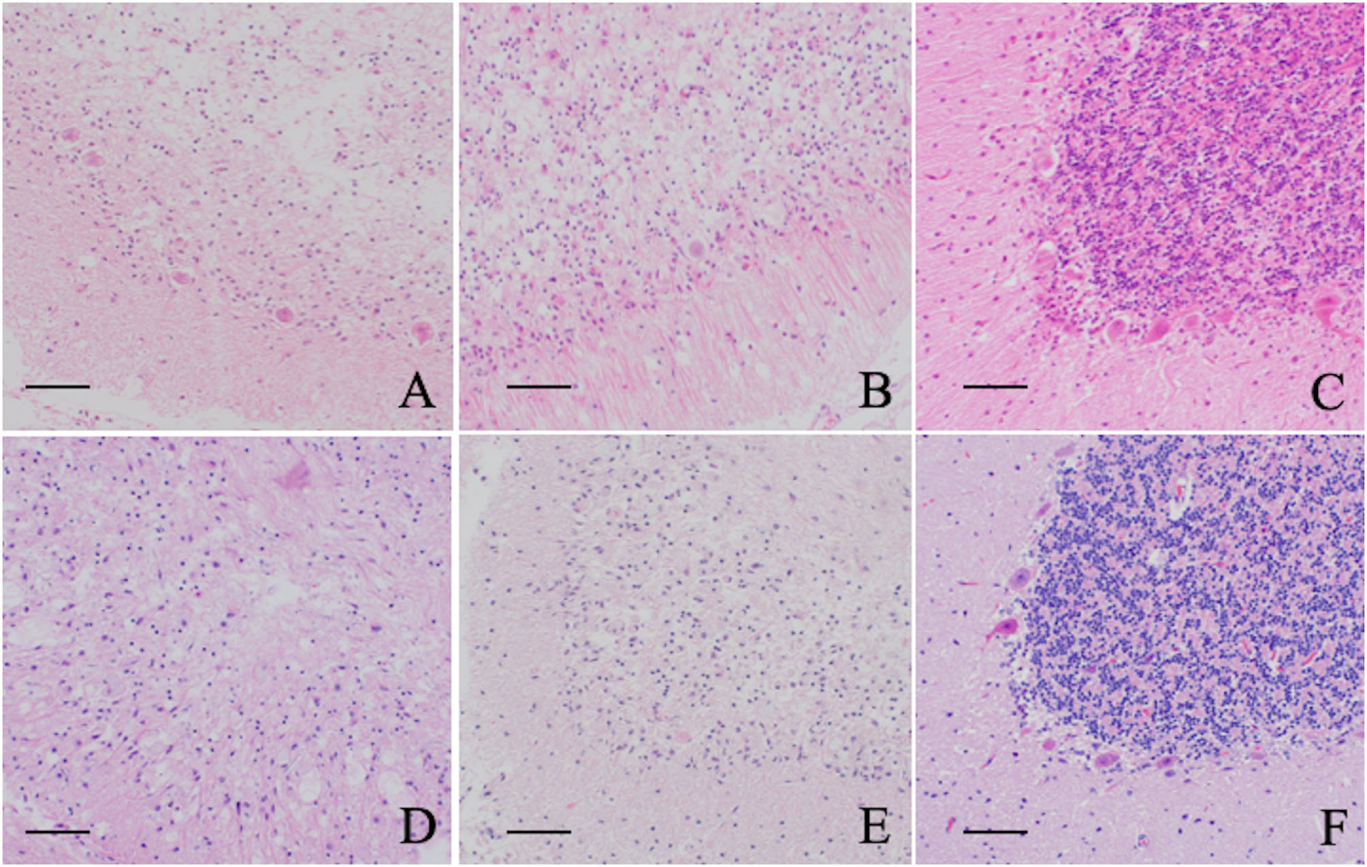
Histopathological features of the cerebellar nodulus and ventral uvula of five lysosomal storage diseases. **(A)** Chihuahuas with NCL (Case 1), **(B)** Border Collie with NCL (Case 3), **(C)** Shikoku Inu with NCL (Case 5), **(D)** Toy Poodle with GM2 gangliosidosis variant 0 (Sandhoff disease) (Case 7), **(E)** Shiba Inus with GM1 gangliosidosis (Case 8), F: normal control dog. Neuronal degeneration and loss are mildly observed in a Shikoku Inu with NCL and severely in other cases compared to a control dog. All sections are HE stained. Bar = 50 μm.

Severe neuronal degeneration and neuronal loss were observed in the NU of Cases 1 (Chihuahua with NCL), 4 (Border Collie with NCL), 7 (Toy Poodle with GM2 gangliosidosis), and 8 (Shiba Inu with GM1 gangliosidosis). Neurons of the molecular, Purkinje, and granular layers are severely decreased throughout the cerebellum, and a variety of intracytoplasmic accumulations were observed in residual Purkinje cells, ceroid-lipofuscin-like materials in Cases 1 and 4, and pale eosinophilic granular materials in Cases 7 and 8, as described in previous reports (7, 10–13, 17). Severe astrogliosis and microgliosis were also observed in the cerebellar cortex. Although neuronal accumulation with ceroid-lipofuscin-like material was observed in the cerebellar neurons of Case 5 (Shikoku Inu with NCL), the pathological extent of neuronal loss appreared milder than in the other cases (Figure 4).

## Discussion

The present hypothesis that not only dogs with NU hypoplasia and tumors affecting NU but also with other diseases, such as LSD, which cause atrophy in the whole brain, may cause NU dysfunction and subsequent PHT, was partially verified in this study. Although cerebellar atrophy was observed in MR images in all cases, details by region in the cerebellum were unclear. However, neuronal degeneration and loss were observed histopathologically in the NU of all diseases in this study, and this is consistent with our previous hypothesis that NU dysfunction causes PHT (1, 2).

PHT observed in the videos of dogs with LSDs discussed in this study was not noticed during consultation (7, 10–13, 17). There were several reasons for this. First, the lack of recognition of this neurological sign until originally described in 2016 is the most common reason. Other reasons include its slightness in some cases, changing the tilt side frequently, and distraction by well-known signs such as intention tremor and ataxia.

The head tilt was slightly to moderate in Chihuahuas with NCL, a Border Collie with NCL, and a Shiba Inu with GM1 gangliosidosis, while it was clearly observed in a Toy Poodle with GM2 gangliosidosis and a Shikoku Inu with NCL. In contrast, neural degeneration and loss were slightly observed in Shikoku Inu with NCL, although severe in other cases. Therefore, the correlation between the degree of head tilt and the pathological severity is unclear. PHT was observed once and then disappeared in Shikoku Inu with NCL in this study. Although the reason was unknown, PHT is reported to be sometimes compensated (1). It may be compensated for its much slower progression in a Shikoku Inu with NCL than in other diseases. Recently, intermittent head tilt was reported in Chihuahua with NCL (9), which may also be PHT.

The limitations of this study include the retrospective review of available videos that were shot without recognition of PHT, the small number of cases, and the inclusion of only dogs. However, it is important that PHT is also observed in some dogs with LSDs. PHT may not be observed in some videos because the dog did not turn the head properly during the recording period. Although PHT was not observed in the videos of the late stage in which the dog could not maintain the trunk position and recumbency, it might be observed in those stages with support to maintain trunk position and turn the dogs' heads side to side as when we evaluate physiological nystagmus. The observers may have been biased when asked to look for an intermittent clinical sign such as PHT in a short video clip. Another limitation include that histopathological changes observed not only in the nodulus and ventral uvula but also whole cerebellum, so it did not confirm the previous suspicion that PHT is related to this specific region. It is unclear whether lesions in other areas of the cerebellum cause PHT.

## Conclusion

PHT was observed not only in dogs with NU hypoplasia and tumors affecting NU reported previously but also in several LSDs in this study. As PHT had not been described until 2016, it might have been overlooked in some veterinary patients. The clinical sign described as “alternating head tilt” in a dog with a tumor, including NU (3), was recognized as PHT (5) only after it was pointed out that it might be PHT (4). Although further observation of many cases with cerebellar diseases that cause NU dysfunction, including vascular diseases, inflammatory diseases, and trauma, is required, PHT may be a common clinical sign and pathophysiology in dogs with NU dysfunction.

## Data Availability Statement

The original contributions presented in the study are included in the article/Supplementary Material, further inquiries can be directed to the corresponding authors.

## Ethics Statement

This is a retrospective observational descriptive study. Therefore, live animals were not used in this study, and the ethical approval is not required due to its nature. However, all owners of cases including this study received an informed consent and had approved that their dogs and data would be used for academic researches. Written informed consent for participation was not obtained from the owners because each case was individually published in papers with written or verbal informed consent at the time. Some of the cases do not have written informed consent, but it is not possible to obtain it because a long time (more than 10 years) has passed and we have not been in contact with each of them since then.

## Author Contributions

ST, YT, YN, and DH participated in clinical case management. MT and KU participated in the pathological examination. ST drafted the manuscript. ST, YN, and DH reviewed the stored videos and MRI data. YT, YN, DH, AY, and OY participated in the review and editing of the manuscript. All authors contributed to the article and approved the submitted version.

## Conflict of Interest

The authors declare that the research was conducted in the absence of any commercial or financial relationships that could be construed as a potential conflict of interest.

## Publisher's Note

All claims expressed in this article are solely those of the authors and do not necessarily represent those of their affiliated organizations, or those of the publisher, the editors and the reviewers. Any product that may be evaluated in this article, or claim that may be made by its manufacturer, is not guaranteed or endorsed by the publisher.

## Supplementary Material

Supplementary Video 1 Edited video of positioning head tilts observed in dogs with lysosomal storage diseases. Chihuahua with neuronal ceroid lipofuscinosis (NCL) (Case 1, 19 months of age), one Border Collie with NCL (Case 3, 16 months of age), one Shikoku Inu with NCL (Case 5, 46 months of age), and one Toy Poodle with GM2 gangliosidosis variant 0 (Sandhoff disease) (Case 7, 18 months of age).

Supplementary Video 2 Shiba Inu with GM1 gangliosidosis (Case 9, 6 months of age).
